# Regulatory Immune Mechanisms in Tolerance to Food Allergy

**DOI:** 10.3389/fimmu.2018.02939

**Published:** 2018-12-12

**Authors:** Pattraporn Satitsuksanoa, Kirstin Jansen, Anna Głobińska, Willem van de Veen, Mübeccel Akdis

**Affiliations:** Swiss Institute of Allergy and Asthma Research, University of Zurich, Davos, Switzerland

**Keywords:** dendritic cells, food allergy, food microbiome, oral tolerance, regulatory T and B cells

## Abstract

Oral tolerance can develop after frequent exposure to food allergens. Upon ingestion, food is digested into small protein fragments in the gastrointestinal tract. Small food particles are later absorbed into the human body. Interestingly, some of these ingested food proteins can cause allergic immune responses, which can lead to food allergy. So far it has not been completely elucidated how these proteins become immunogenic and cause food allergies. In contrast, oral tolerance helps to prevent the pathologic reactions against different types of food antigens from animal or plant origin. Tolerance to food is mainly acquired by dendritic cells, epithelial cells in the gut, and the gut microbiome. A subset of CD103^+^ DCs is capable of inducing T regulatory cells (Treg cells) that express anti-inflammatory cytokines. Anergic T cells also contribute to oral tolerance, by reducing the number of effector cells. Similar to Treg cells, B regulatory cells (Breg cells) suppress effector T cells and contribute to the immune tolerance to food allergens. Furthermore, the human microbiome is an essential mediator in the induction of oral tolerance or food allergy. In this review, we outline the current understanding of regulatory immune mechanisms in oral tolerance. The biological changes reflecting early consequences of immune stimulation with food allergens should provide useful information for the development of novel therapeutic treatments.

## Introduction

**Figure 1:**
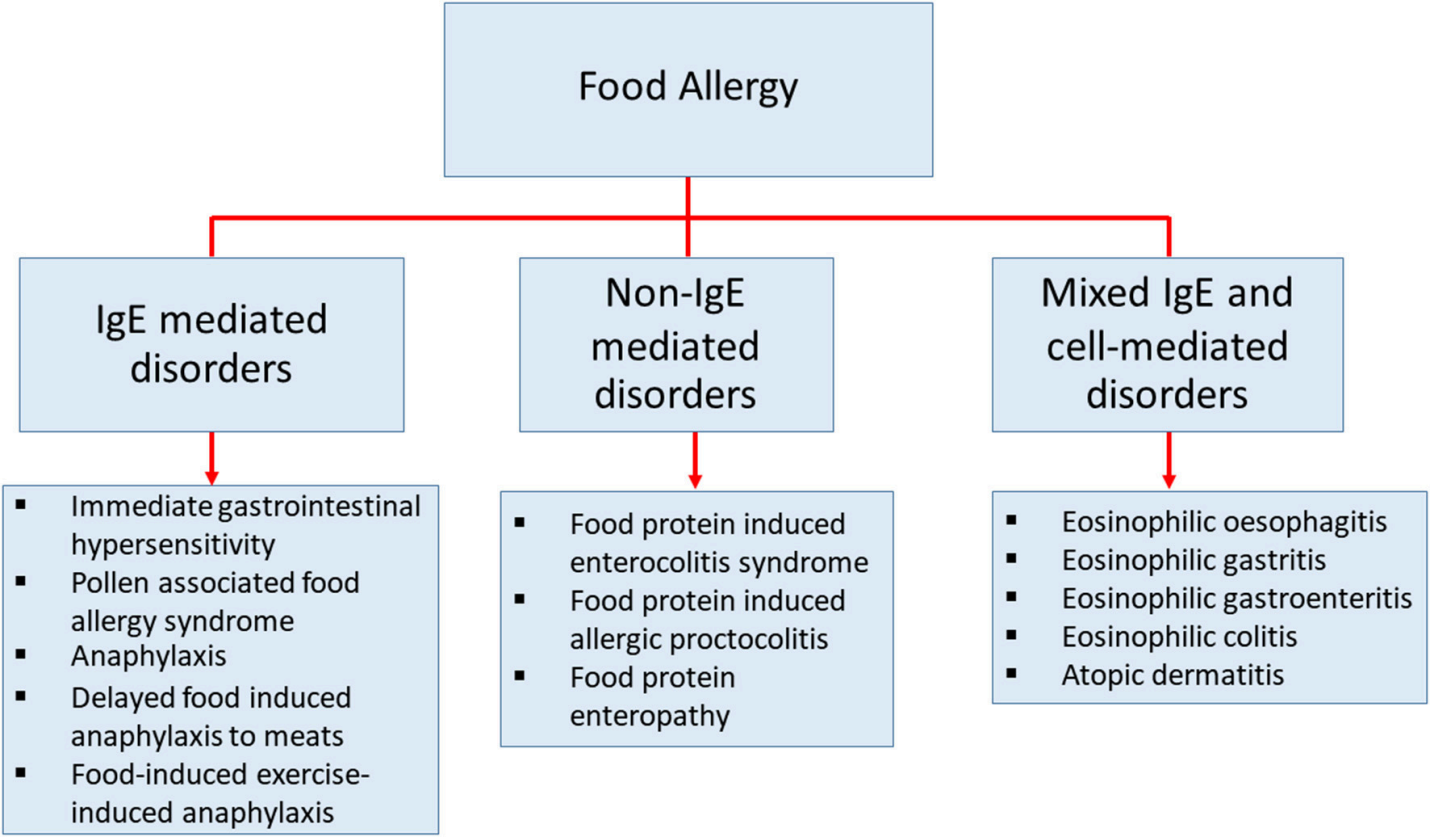
The classification of diseases that cause by food allergy. Classification of food-related diseases by three different immune responses; IgE-mediated immediate disorders, non-IgE-mediated disorders, and disorders with the contributions from mixed IgE-mediated and cell-mediated immune pathways.

Food allergy is defined as an adverse immune response to ingested food proteins. This adverse immune response consists of IgE-mediated immediate hypersensitivity reactions, non-IgE-mediated reactions, and disorders with mixed IgE-mediated and cell-mediated immune reactions (Figure 1) (1–4). Food allergy has become an important public health burden in the past few decades, particularly in developed countries (5–7). The prevalence of food allergies is now estimated at 5–10% of the population in developed countries (8). The prevalence of food-challenge-defined allergies for common food allergens was reported to be: 0.6% to cow's milk, 0.2% to egg, 0.1% to wheat, 0.3% to soy, 0.2% to peanut, 0.5% to tree nuts, 0.1% to fish, and 0.1% to shellfish (9). Besides, the World Allergy Organization provided an extensive study using different approaches of food allergy such as oral food challenge, history of the clinical reaction of food-specific IgE, and food allergy questionnaires in 89 member countries. This study revealed that children from Australia, Finland, and Canada at the age of 5 or lower, have the highest prevalence of food allergy (10). The patterns of food allergy were consistent in many regions showing egg and milk were among the most common allergens in preschool children. In other developed countries, the estimation of overall food allergy prevalence has also increased drastically in the past decades for uncertain reasons (9, 11–14). Therefore, to be able to develop more precise diagnostic approaches, prevention, and medical treatments, a better understanding of the mechanisms in food allergy is necessary (15).

## Induction of Allergic Immune Responses

To know the mechanisms of food allergy, we must understand the role of food allergens in the induction of allergic immune response (16). Food allergens are derived from common naturally-occurring food proteins of plant- and animal-origin (17, 18). The proteins in the food are initially broken down by hydrolytic enzymes in the gastrointestinal track during the digestive process. It is hypothesized that food allergens can be modified into different forms and different structures, which can be processed by antigen presenting cells, exhibited on the major histocompatibility complex class II molecules, and subsequently recognized by antigen-specific T cells (19). The naïve antigen-specific T helper (Th) cells differentiate into effector Th2 cells in the presence of interleukin 4. A set of interleukins such as IL-4, IL-5, IL-10, and IL-13 are massively produced by Th2 cells and induce B cells to differentiate into IgE-producing plasma cells. Antigen-specific IgE antibodies directly bind to high-affinity receptor FcεRI on mast cells and basophils. During the antigen re-exposure, these specific IgE antibodies induce degranulation of mast cells and release of mediators including cytokines, histamine, and proteases which result in allergic symptoms. There are several factors that influence the allergic responses. For example, boiling or frying peanuts can alter the structure of allergens and reduce their allergenicity. The term “allergenicity” is the ability of allergens to initiate a clinical response through an IgE-mediated mechanism (20). The relative amount of major peanut allergen, Ara h 1 is significantly reduced in fried and boiled food preparations, which results in a drastically decreased IgE-binding intensity. Although Ara h 2 and Ara h 3 showed similar relative amounts in fried, boiled, and roasted peanuts, both allergens had lower IgE-binding capacity in boiled and fried peanuts than in roasted peanuts (21). Similarly heating cow's milk and hen's egg, tends to lower allergenicity (22–24).Cow's milk proteins are considered to be the most common food allergens in IgE- and non-IgE-mediated food allergic disorders in children (25). Most individuals with cow's milk allergy are sensitized to caseins and the whey proteins β-lactoglobulin and α-lactalbumin. Caseins are more resistant to high temperatures compared to whey proteins. Cow's milk allergic children who have a high level of casein IgE are less likely to tolerate a baked milk diet compared to those who have a lower level of casein IgE. Specific IgE-binding patterns to casein and β-lactoglobulin peptides could predict the original cause of cow's milk allergy and differentiate subjects between the ones who are more likely to outgrow cow's milk allergy at a younger age vs. and the ones who are more likely to develop allergic symptom at a younger age vs. those with a more persistent cow's milk allergy (26–28). Besides milk proteins, the two major egg allergens, ovalbumin (OVA), and ovomucoid (Gal d1) are major causes of allergy in children. Bloom et al. showed that Gal d1 has higher heat stability than OVA. OVA showed signs of aggregation after 25 min of heating while Gal d1 stayed stable up to 60 min. However, both egg allergens showed strongly reduced IgE-binding capacity after thermal processing. Additionally the presence of wheat during the heating process also affects the allergenicity and reduces the IgE binding further (28). In contrast, the effect of heating shellfish results in increased IgE reactivity to crustacean allergens. A recent study confirmed that a higher level of IgE reactivity was observed in cooked crustacean extracts compared to raw extracts (29).

To date, there is no clear answer to the question of “what makes a dietary or digestible protein an allergen?” Besides, the mechanisms of food allergy development have not been comprehensively described. The allergic reactions to food are expected to be enhanced by the imbalance of immune suppression (30, 31). As a result of the lack of immune suppression, the induction of immune tolerance to food is not achieved in allergic individuals.

## Oral Tolerance

Oral tolerance is the physiological response to ingested antigens. The development of oral tolerance takes place in the gastrointestinal tract. The gut-associated lymphoid tissues play a major role in limiting inflammatory responses to resident bacteria and food proteins (32). To maintain oral tolerance, the gut-associated lymphoid tissues continue to discriminate self from non-self-antigens and recognize the pathogens that can cause tissue inflammation or gut disease. A breakdown in this process occurs when the gut-associated lymphoid tissues fail to perform its functions. Continuously breaking down of oral tolerance leads to the loss of oral tolerance and unwanted allergic responses. (33).There is an enormous unmet need for modern therapeutic treatments to treat patients with food allergy. Therefore, studies dissecting the mechanisms of oral tolerance are very important (34). The possible mechanisms of oral tolerance may involve recognition of food antigens by dendritic cells, robust induction of Treg cells as well as Breg cells. Also, the environment in human gut reinforces and enhances the expansion of the presence of bacteria-derived metabolites and biogenic amines, such as short-chain fatty acids and histamine (35–38).

### Dendritic Cells

Sensitization to food allergens starts with the uptake of antigens in the gut. This occurs through special types of epithelial cells: M cells (39, 40) and more importantly specialized Goblet-cells, called goblet-cell-associated antigen passages (GAPs) (41). Antigens, taken up by M cells or GAPs, can then be transferred to dendritic cells (DCs). In the small intestine, there are two major populations of DCs: CD103^+^CX3CR1^−^ DCs and CX3CR1+ DCs. CX3CR1^+^ DCs are able to directly uptake antigens from the lumen and have more inflammatory potential (42–44). CD103^+^CX3CR1^−^ DCs on the other hand have tolerogenic properties. GAPs exclusively deliver antigens to CD103^+^CX3CR1^−^ DCs and thus are related to the induction of oral tolerance (41).

Besides GAPs, CXCR1+ macrophages can also present antigens from the lumen to CD103+ DCs and induce tolerogenic effects and IL-10 production. (45, 46). After contact with the antigen CD103+CX3CR- DCs can migrate to mesenteric lympnodes in a CCR7 dependent manner (47). In the lympnode CD103^+^CX3CR1^−^ DCs promote the development of T regulatory cells (Treg cells) through the production of TGF-β and RALDH2, an enzyme that converts retinol into retinoic acid (48).

### T Regulatory Cells

T cells play an essential role in food allergies. Th2 cells drive the allergic response by producing IL-4, IL-5, and IL-13. However, other types of T cells play a role in developing tolerance: Treg cells. There are different types of Treg cells, some are thymus-derived and are called natural Treg cells (nTreg cells), and some are induced in the periphery and are called iTreg cells. It was shown by Mucida et al. that oral tolerance can be induced in the absence of thymic-derived Treg cells in a mouse model (49). Besides, Lafaille et al. showed that iTreg cells are essential for establishing oral tolerance (50). The best-described tolerogenic T cells are FOXP3^+^ Treg cells, which are characterized by the expression of CD25 and the transcription factor FOXP3. Treg cells regulate immune responses to allergens through several mechanisms (51, 52). Treg cells can produce different types of inhibitory cytokines, such as IL-10 and TGF-β. Furthermore, they can inhibit antigen-presenting cells by the inhibitory co-stimulators programmed cell dead protein 1 (PD-1) and cytotoxic T-lymphocyte associated protein 4 (CTLA4). Additionally, Treg cells can prevent the proliferation of effector T cells through CD25, a high-affinity receptor of IL-2, by depriving the effector cells of IL-2 (53). Lastly, Treg cells can produce granzyme A and B, which can cause cytolysis of effector cells (54, 55).

Treg cells play a key role in induction and maintenance of tolerance (53, 56). It was shown that FOXP3 knockout mice developed multi-organ inflammatory responses associated with allergic inflammation (57, 58). Adoptive transfer of Treg cells was able to suppress anaphylaxis in a food allergy model in mice and was able to control the Th2 immune response (59, 60). It was shown that children with IgE mediated food allergy have significantly lower FOXP3 expression compared to healthy controls (61, 62) and children with peanut or egg allergy showed a decrease in Treg cell percentage after allergen exposure (63–65). It was also revealed that children with egg allergy have reduced neonatal regulatory T cell function (66). At last, oral immunotherapy, the only known therapy for food allergies, increases Treg cell function, hypomethylation of FOXP3 gene (67) and the number of FOXP3 positive cells (68). In addition to immunotherapy, low dose IL-2 treatment is also able to induce Treg cells and prevent immune responses (69). A combination of the two treatments has been performed by Smaldini et al. and was effective in reversing IgE-mediated food allergy (70).

Besides conventional FOXP3^+^ Treg cells, another type of T-cell that plays a role in oral tolerance is a Th3 cell. Th3 cells do not express FOXP3 or CD25 but express latency-activated peptide (LAP) on their surface, and they are able to produce the inhibitory cytokine TGF-β. It was shown in mice that epicutaneous immunotherapy with OVA-induced Th3 cells that protected against anaphylaxis by suppressing mast cell activation through TGF-β production (71). In an OIT model of cow's milk allergy, fructo-oligosaccharides induced Th3 cells that coincided with protection against anaphylaxis (72). Moreover, Th3 cells were found capable of promoting the development of iTreg cells (73).

TGF-β is not only produced by Th3 cells, but also by conventional FOXP3+ Treg that express LAP and the surface molecule GARP (74). Treg cell expression of GARP is essential for optimal induction of oral tolerance (75). IL-6, IL-21, and IL-35 can inhibit the expression of GARP on FOXP3+ Treg- cells and thereby inhibit LAP and TGF-β. Blocking the IL-6 pathway can enhance oral tolerance in mice (76). Excessive levels of IL-4 also inhibited the induction of allergen-specific Treg cells and caused intestinal inflammation in a mouse model (77).

As mentioned earlier CD103+ DCs produce retinoic acid that is able to amplify TGF-β production and induce FOXP3+ Treg cells (48). Additionally, retinoic acid produced by DCs induces the expression of receptors α4β7 and CCR9 on T cells. These receptors are responsible for T cell homing to the gut (78). It was demonstrated by Hadis et al. that gut-homing and expansion of Treg cells in the lamina propria is necessary to achieve oral tolerance (46). Furthermore, it was shown that homing of IL-10 producing Tregs is important for oral tolerance (79, 80).

Besides the functioning of Treg cells, T cell depletion and anergy can also contribute to oral tolerance. During high dose oral allergen desensitization in cow's milk allergy, a reduction in antigen-specific T cells was observed (81). Additionally, after it was found that during peanut immunotherapy allergen-specific CD4+ T cells can shift toward an anergic Th2 phenotype (82).

### B Regulatory Cells

B cells that can differentiate into antibody-secreting plasma cells are commonly considered to administer immune responses by producing antigen-specific antibodies and help to induce optimal CD4^+^ T cell activation (83). Immunosuppressive Breg cells play a role in the regulation of immune responses by suppressing effector T cells through the production of suppressor cytokines (IL-10, TGF-β, and IL-35) (84). However, the role of Breg cells in oral tolerance remains unclear.

Breg cells moderate immune responses in infection, allergic inflammation, and tolerance, predominantly via IL-10 (85). Van de Veen et al. found that inducible IL-10 secreting B regulatory (Br1) cells contribute to peripheral allergen tolerance in beekeepers. The increment of bee venom allergen-specific, IL-10-producing B cells, has been observed in bee venom allergic patients receiving AIT. Also, Br1 cells produce IgG4 when they switch to plasma cells, which is a non-inflammatory immunoglobulin isotype that prevents IgE-mediated mast cell and basophil degranulation (86). Allergen-specific IgG4 may be involved in promoting immune tolerance in OIT. Santos et al. demonstrated that the ratio of peanut-specific IgG4 to peanut-specific IgE was higher in tolerant patients compared to peanut-allergic patients (87). The essential mechanism of tolerance induction via IL-10-producing B cells was examined with IL-10-overexpressing B cells. IL-10-overexpressing B cells were found to suppress the maturation of dendritic cells, T effector cell proliferation, and IgE production. In addition, IL-10-overexpressing B cells produced the anti-inflammatory IL-1 receptor antagonist and vascular endothelial growth factor and had lower production of pro-inflammatory cytokines (88). Human type 3 innate lymphoid cells (ILC3s) support the maintenance of mucosal tolerance. A recent study showed that human CD40L^+^ ILC3s provide innate B-cell help and are affected in an innate immunoregulatory mechanism through induction of immature translational Breg cell differentiation, which takes place in palatine tonsils *in vivo* (89).

**Figure 2:**
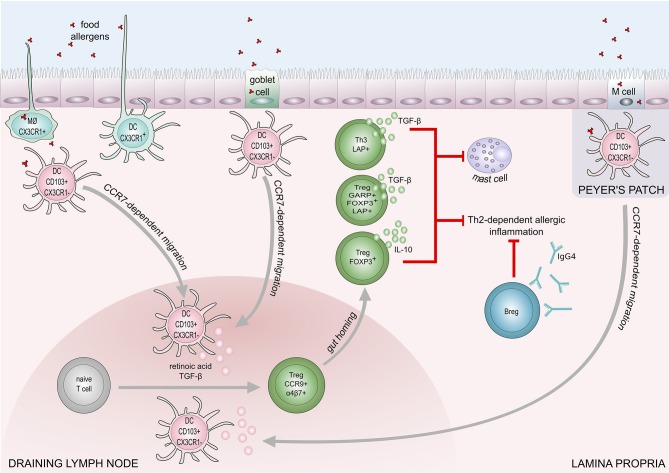
Mechanisms of immune tolerance to food allergens. Induction of food tolerance takes place in the gut when the immune cells encounter food antigens. Several cell types are involved in the antigen uptake: goblet cells, microfold (M) cells, intestinal epithelial cells, CX3CR1^+^ macrophages (MØ), and CX3CR1+dendritic cells. CX3CR1^+^ DCs and CX3CR1^+^ MØ, are capable of extending dendrites to capture antigens on the apical layer of epithelium in the gut lumen. Antigens taken up by CX3CR1^+^ MØ and goblet cells are transferred to CD103^+^CX3CR1^−^ DCs, which subsequently migrate to draining lymph node in a CCR7 dependent manner. Production of retinoic acid and TGF-β foster differentiation of naïve T cells into regulatory T cells (Tregs). Retinoid acid-dependent induction of integrin α4β7 expression on Tregs is responsible for T cell homing to lamina propria. Tregs (Foxp3^+^), and Th3 cells inhibit Th2-dependent allergic inflammation and mast cell degranulation, through the production of IL-10 and TGF-β. Suppression of Th2-responses also engages regulatory B cells (Bregs) that contribute to food tolerance by producing IgG4.

IL-10-producing CD5^+^ Breg cells in mesenteric lymph nodes play a role in the regulation of IgE-mediated anaphylaxis following challenge with cow's milk allergens in a murine model (90). Peanut-specific B cells were increased in the blood after oral immunotherapy in peanut allergic patients (91, 92). A recent study showed IL-10 producing B cells are able to induce and maintain Treg cells in rheumatoid arthritis disease (93). The down-regulation of IL-4, and upregulation of IL-10 production result in an increase of IgG4 and a decrease of IgE levels. IL-10 is known to promote heavy chain immunoglobulin isotype switching to IgG4 while IL-4 induces switching to IgE (94). Furthermore, mucosal IgA inhibits uptake of an antigen by the epithelium and may protect against food allergy (95). The proposed mechanisms of food tolerance are shown in Figure 2.

### Gut Microbiome

The gut microbiome is being increasingly recognized as a major factor in mediating health and disease (96, 97). There are several studies describing the interrelation between microbiota of the gastrointestinal tract, respiratory tract, and skin allergic disorders (98–102). Additionally, it has been shown that the microbiome is associated with oral tolerance (103–105). The human microbiome is capable of inducing Treg cells that suppress Th2-derived responses.

Certain bacterial strains such as *Bifidobacterium longum 35624, Clostridia*, and *Bacteroides fragilis* can induce intestinal Treg cells that are able to suppress food allergy and colitis (106, 107). Pattern-recognition receptor activation on DCs is a potential mechanism by which intestinal microbes (Lactobacillus rhamnosus JB-1) may promote Treg cell differentiation (108). A study from the National Institutes of Health, Human Microbiome Project revealed 14 important bacterial strains by sequencing and analytical processing 380 whole-genome shotgun samples (109). In addition, a 16S rDNA gene was sequenced to characterize the oral bacterial composition in saliva samples from healthy and allergic children up to 7 years of age. The result affirmed that early changes in oral microbial composition seem to influence immune maturation and allergy development (110, 111).

The potential role of the gut microbiome in food allergy has been studied in mouse models. Rodriguez et al. demonstrated that intestinal colonization of *Staphylococcus* protects against oral sensitization and allergic response in a mouse model. This was the first study to describe a relationship between alterations within the subdominant microbiota and severity of food allergy (112). Another study showed that allergen-sensitized (Il4ra^F709^) mice had a different microbial composition compared to wildtype mice with an increased abundance of different bacterial families including Lachnospiraceae, Lactobacillaceae, Rikenellaceae, and Porphyromonadaceae. This different microbial composition increased OVA-specific responses and anaphylaxis when reconstituted in wild-type germ-free mice, which indicates that the microbial composition play a role in food allergy (113).

Rivas et al. demonstrated that disease-susceptible (Il4ra^F709^) mice with an enhanced interleukin-4 receptor (IL-4R) signaling exhibited STAT6-dependent impaired generation and function of mucosal allergen-specific Treg cells. Their study showed that the Treg cells failed to suppress mast cell activation and expansion (114). Those Treg cells were reprogrammed into Th2-like cells and participated in the development of food allergy (115). Another study determined that microbiota regulates Th2 responses through the induction of RORγt Treg cells and Th17 cells (116).

Moreover, the bacterial metabolites, such as short-chain fatty acids (SCFA's) and biogenic amines produced in the human gut play a role in host immune regulatory activity (117, 118). SCFA'sare able to enhance dendritic cell regulatory activity, leading to the induction of Treg cells and IL-10-secreting T cells (119). SCFA's can be produced by bacteria after digestion of dietary fibers. It was shown that infants with a diet consisting of high levels of fruits and vegetables is associated with less food allergy by the age of 2 years, which could be due to an increased amount of dietary fiber intake (120). It was shown in mice that deficiency of dietary fiber intake increases the susceptibility to OVA induced allergic airway inflammation (121). Additionally, it was shown that uptake of polyunsaturated fatty acids can increase the production of SCFA's by bacteria (122) and the dietary intake of poly unsaturated fatty acids was inversely associated with atopy in childhood (123).

The secretion of biogenic amines such as histamine has extensive effects on many immune cell types (124, 125). Histamine levels are increased in patients with irritable bowel syndrome, inflammatory bowel disease and in adult asthma patients (126, 127).

Food allergy could be related to changes in microbial exposure in early life, which affect host microbiota composition, modifies the development of host immunity, and causes pathogenic immune responses to food allergens (96). How the microbiome exactly affects food allergy should be further investigated.

## Conclusion

Loss of oral tolerance can lead to the development of food allergy in children and adults. However, the development of food allergy in terms of molecular and cellular mechanisms has not yet been demonstrated. The induction or loss of oral tolerance is likely modulated by the combination of DCs, Treg cells, Breg cells, and microbiome. DCs are capable of inducing Treg cells, which produce anti-inflammatory cytokines and are able to suppress T effector cells. Additionally, Breg cells can produce anti-inflammatory cytokines as well and can produce IgG4, which is the anti-inflammatory Ig isotype. So far food allergies are mainly managed by strict avoidance of the food allergens and can only be treated with immunotherapy. How immunotherapy exactly works is not entirely understood. Therefore, the underlying mechanisms of induction and loss of oral tolerance need to be more clearly identified so novel therapeutic treatments can be developed.

## Author Contributions

PS, KJ, AG, WvdV, and MA prepared the manuscript.

### Conflict of Interest Statement

The authors declare that the research was conducted in the absence of any commercial or financial relationships that could be construed as a potential conflict of interest.

## Abbreviations

Breg: Regulatory B cell
DC: Dendritic cell
GAP: Goblet-cell-associated antigen passages
LAP: Latency-activated peptide
OIT: Oral immunotherapy
OVA: Ovalbumin
SCFA: Short chain fatty acids
Treg: Regulatory T cell.
